# Understanding the Goals of Everyday Instrumental Actions Is Primarily Linked to Object, Not Motor-Kinematic, Information: Evidence from fMRI

**DOI:** 10.1371/journal.pone.0169700

**Published:** 2017-01-12

**Authors:** Toby Nicholson, Matt Roser, Patric Bach

**Affiliations:** School of Psychology, University of Plymouth, Drake Circus, Devon, United Kingdom; University of Bologna, ITALY

## Abstract

Prior research conceptualised action understanding primarily as a kinematic matching of observed actions to own motor representations but has ignored the role of object information. The current study utilized fMRI to identify (a) regions uniquely involved in encoding the goal of others’ actions, and (b) to test whether these goal understanding processes draw more strongly on regions involved in encoding object semantics or movement kinematics. Participants watched sequences of instrumental actions while attending to either the actions’ goal (goal task), the movements performed (movement task) or the objects used (object task). The results confirmed, first, a unique role of the inferior frontal gyrus, middle temporal gyrus and medial frontal gyrus in action goal understanding. Second, they show for the first time that activation in the goal task overlaps directly with object- but not movement-related activation. Moreover, subsequent parametric analyses revealed that movement-related regions become activated only when goals are unclear, or observers have little action experience. In contrast to motor theories of action understanding, these data suggest that objects—rather than movement kinematics—carry the key information about others’ actions. Kinematic information is additionally recruited when goals are ambiguous or unfamiliar.

## Introduction

Action goal understanding lies at the heart of social interaction. Understanding *why* people behave as they do allows one to attribute mental states to them, predict what they will do next, and to coordinate one’s own behaviour with theirs [1–3]. Humans make such inferences readily and fluently, even though goal information is often not directly available from the stimulus and requires going beyond the information given, often several steps into the future (e.g. that grasping a cookie ultimately serves the goal of eating, [4]). Since the discovery of mirror neurons in the premotor and parietal cortices of the macaque, it has been argued that the human capacity to derive such task goals may rely on a process that ‘directly matches’, based on available kinematic information, the observed action to an action in their own motor repertoire, allowing it to be identified and its goal to be derived [4–6]. However, even though it is now clear that human action observation similarly engages neuronal ensembles involved in action execution [7–12], attempts to link these activations to goal understanding have been less successful [13]. A growing number of studies link action understanding to regions outside the classical parietal-premotor “mirror” networks, such as the medial prefrontal cortex, the superior temporal sulcus, or the posterior temporal lobe (e.g. [14–17], for meta-analysis, see [18]). Since then, research interest has shifted towards characterizing this extended “action observation network” rather than the core mirror regions alone. Even key proponents of the mirroring view now agree that mirroring may be only one of several action understanding mechanisms [19–20], while others suggest it to be a secondary step after initial goal inferences have been made, for example, to verify that the predicted action is indeed occurring [21–23].

This raises the question on what, if not motoric information, could action goal understanding then be based? Recently, we and others (e.g., [23–27]) have argued that objects would play a major role. The effective use of objects is a defining feature of human action. From early on, humans represent objects teleologically, not only in terms of how they have to be used, but also in terms of their function: the goals that can be achieved with them [26–30]; for a review, see [31, 32]. A tap, for example, is *for* getting water, a hammer is *for* driving in nails, and a credit card is *for* paying. This knowledge about functional object use is supported by dedicated (and evolutionary recent) brain systems in the left hemisphere, spanning prefrontal, parietal and occipitotemporal cortices [33–34]. Lesions to these areas not only impair knowledge of how objects have to be manipulated for successful action [35] or how they can be mechanically combined with one another [32], but also knowledge about the goals they help to achieve—what the objects are ‘for’ [35–36]. We [23] and others [22, 25, 27] have argued that this function knowledge could make a major contribution to action goal identification, in many cases over and above kinematic or motor information. Consider the actions of inserting a letter into a letterbox or inserting a credit card into a cash machine. The actions’ kinematics are virtually identical in both situations, and only indicate, perhaps, that an act of insertion takes place. Knowledge about the objects’ function, however, provides direct access to the task goals that could be achieved in the two situations (in adults: [23, 37], children: [26–27]). In such object-based views of action understanding, kinematic or motor information would only come into play after such initial goal inferences have been made, allowing observers to ascertain which of several possible actions with an object is currently carried out ([23, 25, 38]; for similar arguments, see [21–22]).

The current study was designed as a direct test of these object-based views of action goal understanding. Prior research has suggested that, when stimuli and tasks are well controlled, there is little overlap between understanding higher level action goals and the classical premotor-parietal “mirror” networks [14–15]. Object-based views of action understanding predict instead that the brain regions recruited during action goal identification should overlap to a large extent with brain regions involved in object identification and encoding of object semantics. Here, we therefore directly compared, for the first time, goal identification with kinematic/motor processing of the action on the one hand and object-based processing on the other, within the same study and using identical stimuli. We tested which brain regions are uniquely involved in action goal understanding, and which ones are shared with either object or motor/kinematic processing.

Participants watched videos of a wide variety of everyday object-directed actions. Their task was manipulated to engage either brain regions involved in movement, object or goal identification, while visual stimulation was kept identical. First, the *movement task* was designed to specifically engage regions involved in the motoric/kinematic representation of actions. Participants were instructed to track the kinematics of the hand movements involved in the action and—in order to ensure that they actively engaged with this task—to press a button on the rare occasion (5.5% of all movies) that two consecutive actions involved the same hand movements (e.g., screwing in a light bulb, screwing a lid on a bottle). To ensure goal or object processing would not incidentally contribute to the task, movement repetitions always involved different objects as well as goals and participants were told that they could ignore these action aspects. Second, the *object task* was designed to activate regions involved in object identification and the representation of object semantics. Participants pressed a button in the rare case (5.5% of movies) that two actions involved the same type of object. To ensure that the object task would only require object identification—and not goal or movement processing—participants were told to ignore these action aspects and object repetitions always involved different movements and different goals (e.g., spoon used to sprinkle sugar on a cereal, to stir coffee). Third, and finally, the *goal task* was designed to engage regions involved in action goal understanding. Participants responded whenever two consecutive actions satisfied the same goal (e.g., illuminating a room by pressing on a light switch, or by opening the blinds, again 5.5% of cases), while ignoring object and movement repetitions.

Similar 1-back designs have been used before to ensure participants maintain continuous attention on relevant task aspects (e.g., various localiser task, e.g., [39–41]) and reliably separate the brain regions associated with different task components (see [42] for a meta-analysis in the domain of working memory). Here, this design allows us to test, first, whether there are regions uniquely involved in encoding action goals, rather than the movements performed or the objects used. Such regions should be more strongly activated in the goal task than in either object and movement task. Identifying such regions is important because prior studies searching for regions involved in action goal identification typically only controlled for one of the two factors—object or movement—but not both, and object semantic tasks in particular typically activate similar regions as action understanding tasks (e.g. inferior frontal and middle temporal regions), raising the possibility that goal activations actually reflect object processing for example.

Second, and more importantly, this design allows us to directly test to what extent goal understanding involves—next to regions uniquely sensitive to action goals—brain regions involved in either object or kinematic processing of the actions, or both. To the extent that goal understanding is based on motoric matching (as proposed by mirroring approaches), the goal task should share activation with the movement task, when object processing is controlled. Conversely, if the goal task draws upon object knowledge (as proposed by object-based models of action understanding), then it should share activation with the object task (if movement processing is controlled), specifically temporal and inferior frontal regions typically involved in processing information about object semantics or function. After the experiment, all participants rated the actions they saw with regard to (a) how apparent the goal was, and (b) how much experience they had with the actions. These ratings allow us, to parametrically link, on single subject level, identified activations to their subjective “meaning” of the participant.

## Method

### Participants

Fifteen students at the Plymouth University (4 males, mean age = 25, SD = 7.5, right handed, with normal vision) provided written consent to take part in the experiment. They were recompensed £8 per hour, plus a flat fee (£14) for travel expenses. The study was approved by Plymouth University’s Faculty of Health and Human Sciences Research Ethics Committee prior to data collection. Sample size was determined by prior studies in the field using similar paradigms (e.g., [38]). One participant was excluded because she reported after scanning that she had misinterpreted one of the tasks. Exclusion/inclusion of this participant does not qualitatively alter the results.

### Stimuli

48 different movies (duration 3 seconds each) made up the stimulus set (see supplementary materials). All showed common everyday instrumental actions that were carried out with the hands. The actions were selected such that no other effectors were involved. Moreover, all clips were shot such that the immediate action goals were achieved within the clip. For example, scraping egg on to a plate supports the goal of transferring egg and the higher-level goal of preparing lunch. However, only the low-level goal of egg transfer is fully achieved by the presented action. The 48 actions were subdivided such that there were 8 pairs (16 movies) of actions that involved the same movement in each pair, while goals and objects were different (e.g., screwing in a light bulb, screwing the lid of a jar). In another 16 movies, the tools used in each pair were of the same type, while goals and movements differed (e.g., using a spoon to stir, using a different spoon to sprinkle sugar). In the last remaining 8 pairs, the actions in each pair subserved the same goal, while the movements and objects used were different (e.g., paying with a credit card, paying with paper).

### Tasks

**Fig 1:**
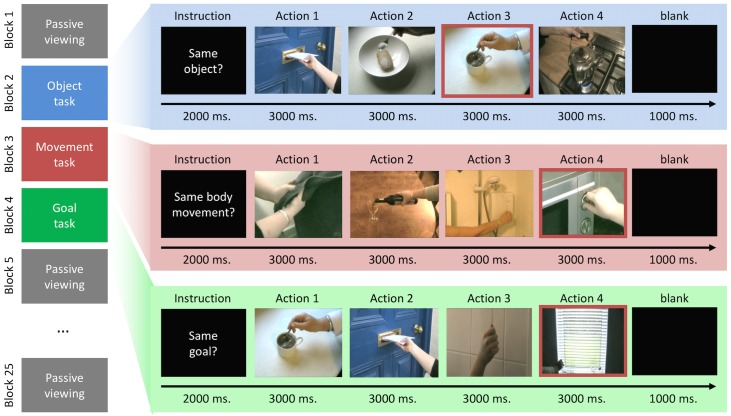
Behavioural task. Figure 1 presents a schematic image of the behavioural task. The left hand side of the image illustrates the (fully counterbalanced) sequence of the mini-blocks/tasks across a run (passive viewing baseline, object task, movement task and goal task). On the right, an example of an experimental mini block for each condition is depicted. Note that the image with a red border within each mini-block illustrates a repetition for that condition (e.g. a repeated object, movement or goal).

There were four tasks, which varied between blocks, and which were assumed to preferentially engage regions involved in processing the three main action aspects (see Fig 1). First, in the ‘goal task’, participants had to press a button when two consecutive actions subserved the same goal (e.g., paying by inserting a credit card, or paying by giving money), but to ignore repetitions of movements or objects. Goals were defined as the immediate action goals that would be achieved by the presented actions. Second, in the ‘movement task’, participants responded when two consecutive actions were presented that involved the same manual movements (e.g., screwing in a light bulb, screwing on a lid of a bottle), but ignored repetitions for objects and goals. Third, in the ‘object task’, participants responded when two actions involved the same general type of object (e.g., using a spoon to stir or to sprinkle sugar on cereal), but ignored repetitions of goals or movements. In both the object and movement task we ensured that the tasks did only draw on processing of object and movement information, not on action goal information. Thus, in each object repetition, the objects were used for different goals and manipulated differently, and in movement repetitions, the movements were performed to achieve different goals and involved different objects. Finally, the ‘passive viewing task’ served as common baseline for the three tasks. Here, participants merely observed the clips and did not respond. Please note that each task was performed on exactly the same 48 action clips. Any differences in brain activity must therefore be due to task rather than stimulus differences.

### Procedure

Before entering the scanner, participants were shown an example of a mini-block (consisting of 4 clips) for each experimental condition (goal, object, and movement) and asked to report where, if at all, the repetition occurred. A repetition was placed within each example but its position in the sequence varied. All participants detected all repetitions.

Participants then performed 6 scanning runs of the experiment, with the order being counterbalanced across participants. Each run started and ended with 15 seconds of blank screen (not analysed), and consisted of 25 mini-blocks, each 15 seconds long. The first and every fourth mini-block thereafter was the ‘passive viewing’ (baseline) task. The three mini-blocks between these baseline blocks always contained all three experimental conditions (movement, object, goal). Their order was pseudo-randomised, such that each possible order of the three tasks (movement, object and goal) occurred equally often in each scanning run.

At the start of each mini-block, a 2000ms instruction informed the participant about the relevant type of repetition that they had to report (e.g. same bodily movement, same object, same goal or just observe/baseline). Then four clips were shown, each lasting three seconds. The instruction remained on the screen underneath each clip. Participants pressed a button as soon as they noticed a relevant repetition. A mini-block ended with one second of blank screen, resulting in a total block duration of 15 seconds.

The order of clips was pseudo-randomized such that each of the 48 clips was presented twice throughout each scanning run (twelve times for the whole experiment), and occurred equally often in each task. The repetitions that participants had to detect were rare (5.5% of the movies in each task) and served as catch trials. Each of the 24 different repetitions occurred only once throughout the experiment and equally often in each task (8 goal, 8 object, 8 movement). Repetitions were organised such that none of the goal tasks contained movements or objects repetitions, none of the object tasks contained goal or movement repetitions, and none of the movement tasks contained object or goal repetitions. The order of the two clips in each repetition was counterbalanced across participants, so that half the participants saw them in one order and the other half saw them in the opposite order.

After the experiment, all participants filled out two questionnaires, one measuring how often they had performed each action and one measuring how apparent the goal of each action was (see also [38, 43–44]). For each of the 48 actions, a representative still frame was shown next to a seven point Likert scale. For the questionnaire measuring goal apparentness, they noted how apparent the goal of the action was on a 7 point Likert scale, anchored with “not at all” and “very clear” at the two ends. For the questionnaire measuring sensorimotor experience, they indicated how often they had previously performed this action, anchored by “not at all” and “very often”.

### Data acquisition and analysis

Data was acquired on a 1.5T Phillips MRI scanner, equipped with a parallel head coil. An echo-planar imaging sequence was used for functional imaging (time repetition (TR) = 3000ms, time echo = 45ms, flip angle = 90°, and field of view = 240, 34 axial slices, 96 x 96 in-plane matrix, 3.9mm slice thickness). Functional data was recorded with 2.5 x 2.5 x 3.9 mm, 0 mm gap, resolution and resampled to isometric 3 x 3 x 3 mm voxels with trilinear interpolation. The whole of the cortex and large parts of the cerebellum were scanned. BrainVoyager QX 2.4 was used for pre-processing and statistical analysis. Functional data was motion and slice time corrected and low-frequency drifts were removed with a temporal high-pass filter (0.006 Hz.). Runs with residual motion artefacts were not considered for analysis (less than 8% of the data). A Gaussian kernel (6-mm full width at half-maximum) was used to spatially smooth the data. Functional data was manually co-registered with 3D anatomical T1 scans (1 x 0.9 x 0.9 mm resolution). The anatomical 3D scans were transformed into Talairach space and the parameters for this transformation were subsequently applied to the co-registered functional data.

In order to generate predictors for the multiple regression analyses, the event time series for each condition were convolved with a two gamma HRF (onset of response and undershoot, 5s and 15s respectively, undershoot dispersion 1, response to undershoot ratio 6). Four predictors of interest were created, three to model the three experimental conditions (Object, Movement and Goal tasks) and one to model the button presses of the participants, capturing the explicitly detected repetitions in each task and the associated motor responses. Voxel time series were z-normalized for each run, and additional predictors accounting for baseline differences between runs were included in the design matrix. The regressors were fitted to the MR time series in each voxel. All reported whole-brain analyses were initially thresholded at *p* < .005, and whole-brain corrected for multiple comparisons using Brain Voyager’s cluster threshold estimator plug-in. This uses a Monte Carlo simulation procedure (1,000 iterations) to establish the critical cluster size threshold corresponding to a family-wise alpha of 0.05 corrected for the whole brain. Only clusters exceeding this threshold are reported.

For the parametric trial-by-trial analyses, the data from the post-scan questionnaires were used to correlate, in separate analyses, brain activity in the experimental tasks to both the apparentness of the goal in each action and how often the actions had been performed, on a single subject level. Some participants had notified us that they found a very small number of actions hard to identify visually, and that they had indicated this by very low ratings on the apparentness questionnaire. We therefore excluded, on a single subject basis, ratings for actions, which were over 3 standard deviations below the mean rating of each action for this participant. This led to the exclusion of less than 3% data points for the parametric analyses, from both the ‘apparentness’ and the ‘how often performed’ questionnaire.

Each subject’s remaining ratings was then utilized to construct eight parametric predictors for the event series. To this end, each participant’s ratings of the 48 actions were transformed to z-scores. Eight predictors were then created, for each participant separately, each modelling parametrically the z-transformed ratings for either goal apparentness or sensorimotor experience, separately for each of the four tasks (object, movement, goal, baseline). We chose eight regressors to model these ratings in a task specific way, because it is likely that the two action aspects—sensorimotor experience and goal apparentness—are differentially relevant in different tasks. As these predictors were statistically independent of one another they can be effectively combined to for maximum power to identify variations that are constant across tasks for a fully corrected whole brain analysis, but also interrogated separately to find potential task-specific effects (in a more restricted region of interest analysis, based on the regions identified in the statistically independent main analysis). Additional predictors for the different mini blocks (goal, object, movement) were added to model any overall differences between tasks, and another additional predictor was used to model responses to the excluded items.

## Results

### Behavioural data

For analysis of response times, we calculated, for each participant and each task, the average time it took participants to identify a relevant repetition, relative to the onset of the repeated movie. Trials with negative response times or below 200 ms. (indicating a false alarm to a prior movie) were excluded. A three-level repeated measurements ANOVA (goal, movement, object) did not reveal any significant differences, *F*[2,26] = .677, *p* = .506 (Greenhouse-Geisser corrected). Participants took similar amounts of time to identify movement (*M* = 1591 ms.), object (*M* = 1647 ms.), or goal repetitions (*M* = 1516 ms.), arguing against different perceptual strategies or attention sets across tasks.

Accuracy data confirmed this interpretation. We first tested whether participants were generally more likely to respond in one of the tasks (irrespective of whether the sequence contained a repetition or not). We calculated the relative frequency of button presses in each condition, across the analysed scanner runs. Numerically, participants, on average, made slightly more button presses in the movement task (M = 37%) than the goal (M = 32%) and object tasks (M = 32%), but this difference was not significant, *F*[2,26] = 2.513, *p* = .120. Second, to test whether the conditions differed in the ease with which a repetition could be detected, we calculated each participant’s ability to detect a repetition as the difference between the percentage of trials in which a repetition was correctly detected (‘hits’) and the percentage of trials in which participants falsely reported a repetition (‘false alarms’). Numerically, the increase in button presses for sequences with a repetition relative to sequences without a repetition was higher in the goal (M = 73%) than the movement (M = 69%) and object tasks (M = 60%), but these differences were not significant, *F*[2,26] = 1.643, *p* = .215, arguing against systematic differences between the tasks.

### fMRI main contrasts

**Fig 2:**
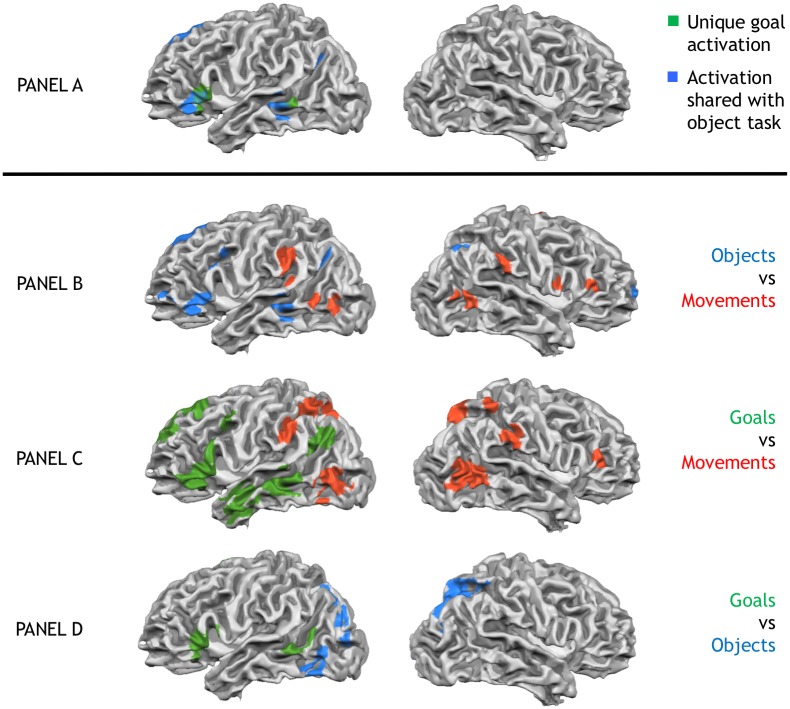
Unique goal activations & pairwise comparisons of goal, movement and object tasks. Panel A shows unique activation in the goal task (green), as well as activation shared between goal task and object task (blue). No activations were shared with the movement task. Panel B to D show pairwise comparisons of the three tasks. Panel B: Object task (blue) vs. Movement task (red). Panel C: Goal task (green) vs. Movement task (red). Panel D: Goal task (green) vs. Object task (blue). All activations thresholded at *p* < 0.005 and whole-brain corrected to a familywise error *p* < 0.05.

To ensure that our movement and object tasks indeed replicated the expected activations, we directly contrasted activity in both tasks against one another (see Table 1, Fig 2, panel B). As expected, the movement task activated regions typically found in action observation/mirror neuron studies, such as the inferior parietal lobe/supramarginal gyrus bilaterally and the right superior and inferior frontal gyri, as well as motion and body sensitive regions in the posterior temporal lobe [45–46]. In contrast, the object task activated a number of left hemispheric regions found in object semantics tasks, such as the inferior, middle and superior frontal gyri, as well as areas in the middle temporal gyrus and fusiform gyrus. In particular, we replicate the distinction between the left and right premotor cortices being primarily involved in object or movement representation, respectively [47].

**Table 1:** Pairwise comparison of the goal, movement and object task.

**Contrast**	***Comparing Object and Movement tasks***				
	**Region (BA)**	**R/L**	**x,y,z**	**t**	**mm**^**3**^
Object > Movement	Cerebellum	R	27,-79,-30	7.70	3024
	Middle Temporal Gyrus/Superior Temporal Sulcus (21)	L	-60,-37,-2	7.51	2727
	Angular Gyrus (39)	L	-30,-65,32	6.17	2295
	Fuisform Gyrus (37)	L	-27,-37,-17	4.74	1728
	Inferior Frontal Gyrus (47)	L	-50,34,-5	11.19	1485
	Parrahippocampal Gyrus (30)	L	-11,-49,5	5.93	1458
	Inferior Parietal Lobe (19)	R	31,-69,41	6.41	1404
	Inferior Frontal Gyrus (10)	L	-42,49,0	6.29	1242
	Middle Frontal Gyrus (9)	L	-51,18,26	5.06	1188
	Superior Frontal Gyrus (8)	L	-12,38,52	4.64	999
	Middle Occipital Gyrus (18)	L	-29,-87,2	4.78	756
Movement > Object	Inferior Parietal lobe /Supramarginal Gyrus (40)	R	60,-44,23	6.28	2376
	Inferior Parietal lobe /Supramarginal Gyrus (40)	L	-59,-40,35	5.42	1971
	Middle Temporal Gyrus (37)	R	52,-55,-2	4.93	1728
	Middle Occipital Gyrus (19)	L	-51,-68,-5	6.13	1620
	Cerebellum	R	21,-44,-34	5.40	864
	Superior Frontal Gyrus (6)	R	17,-9,70	4.99	837
	Inferior Frontal Gyrus (46)	R	49,30,11	4.67	756
**Contrast**	***Comparing Goal and Movement tasks***				
Goal > Movement	Middle Temporal Gyrus/Superior Temporal Sulcus (21)	L	-60,-42,-1	10.11	11502
	Inferior Frontal Gyrus (47)	L	-48,31,-4	13.24	10476
	Superior/Medial Frontal Gyrus (9)	L	-14,48,31	7.43	6021
	Angular Gyrus (39)	L	-35,-64,31	6.60	4833
	Cerebellum	R	18,-69,-29	6.17	3348
	Posterior Cingulate (30)	L	-11,-55,11	7.34	1755
	Posterior Cingulate (31)	L	-12,-49,28	6.12	1620
	Fuisform Gyrus (37)	L	-29,-39,-18	6.07	1404
	Medial Frontal Gyrus (10)	L	-5,50,-4	8.12	1296
	Medial Frontal Gyrus (6)	L	-6,3,60	7.48	1242
	Cerebellum	R	36,-60,-29	7.17	783
	Superior Frontal Gyrus (10)	R	31,50,13	5.49	783
	Fusiform Gyrus (37)	R	18,-42,-8	4.65	702
Movement > Goal	Inferior Temporal Gyrus (37)	R	46,-69,1	8.61	4968
	Inferior Temporal Gyrus (37)	L	-44,-68,-1	9.50	4077
	Inferior Parietal Lobe (40)	L	-50,-39,33	5.56	3672
	Superior Parietal Lobe (7)	R	10,-67,44	6.44	1647
	Inferior Parietal Lobe /Supramarginal Gyrus (40)	R	57,-36,37	5.03	1188
	Middle Frontal Gyrus (46)	R	43,32,16	5.57	1080
	Superior Parietal Lobe (7)	L	-20,-67,43	5.04	891
	Inferior Parietal Lobe (40)	R	36,-46,53	5.28	783
**Contrast**	***Comparing Goal and Object tasks***				
Goal > Object	Inferior Frontal Gyrus (45)	L	-53,21,5	6.36	4320
	Middle Temporal Gyrus (39)	L	-48,-58,13	4.99	2403
	Superior Frontal Gyrus (6)	L	-6,-4,62	9.56	2349
	Anterior Cingluate Gyrus (32)	L	0,26,24	4.92	1242
Object > Goal	Superior Parietal Lobe (7)	R	28,-65,49	7.07	5562
	Superior Parietal Lobe (7/19)	L	-26,-69,31	6.80	2997
	Inferior Temporal Gyrus (37)	L	-48,-67,-4	5.66	2160
	Lingual Gyrus (18)	L	-2,-86,2	5.34	1971

Having established that the object and movement tasks activate typical regions for movement and object representation, our first goal was to identify regions selectively involved in encoding action goals rather than object semantics or movement kinematics. We therefore ran a conjunction analysis to find regions with stronger activation in the goal task than either the movement or object tasks (goal > movement ∩ goal > object). This analysis revealed three left hemispheric regions, the inferior frontal gyrus, middle temporal gyrus/superior temporal sulcus and the medial frontal gyrus, which were specifically involved in the goal task relative to both, the movement and object task (see Fig 2, panel A, green, Table 2).

**Table 2:** Conjunction analyses. Shared and unique activation in the goal task.

**Contrast—*Unique activation in the goal task (Goal > Movement ∩ Goal > Object)***				
**Region (BA)**	**R/L**	**x,y,z**	**t**	**mm3**
Inferior Frontal Gyrus (45)	L	-51,23,6	6.37	2889
Middle Temporal Gyrus/Superior Temporal Sulcus (22)	L	-58,-42,3	4.02	648
Medial Frontal Gyrus (6)	L	-5,2,61	5.86	567
**Contrast—*Shared activation of goal and object task (Goal > Movement ∩ Object > Movement)***				
**Region (BA)**	**R/L**	**x,y,z**	**t**	**mm3**
Middle Temporal Gyrus/Superior Temporal Sulcus (21)	L	-60,-37,-2	6.95	2403
Cerebellum	R	24,-76,-26	4.92	1566
Inferior Frontal Gyrus (47)	L	-48,30,-2	8.58	1485
Middle Frontal Gyrus (9)	L	-39,8,37	4.42	675
Angular Gyrus (39)	L	-33,-64,34	4.89	594
**Contrast—*Shared activation of goal and movement task (Goal > Object ∩ Movement > Object)***				
*no activations passed whole brain-correction*				

Our second goal was to establish whether the goal task, next to these uniquely activated regions, would additionally recruit object and movement selective regions. We therefore tested, in separate conjunction analyses, to what extent the goal task shared activations with either the object or movement task. First, to find overlapping activation with the object task, we calculated the conjunction of regions that were more strongly activated in object than the movement task and more strongly activated in the goal than the movement task (goal > movement ∩ object > movement). This contrast therefore identifies shared activation of the goal and object tasks while movement processing is controlled. It revealed that core regions of the object network identified in our study were also activated in the goal task: the middle temporal gyrus/superior temporal sulcus, the inferior frontal gyrus, and the cerebellum (Table 2, Fie 2 –panel A, blue). Thus, goal and object task share overlapping activation, as predicted from the idea that action goal identification draws upon information about object function (e.g., [22–23]).

The opposite conjunction, identifying regions more activated in the goal and movement tasks relative to the object tasks (goal > object ∩ movement > object), did not reveal any significant activation. This indicates that the goal task does not activate any of the regions engaged by the movement task, when object-related activations are controlled. To further confirm this interpretation, we conducted a whole-brain direct comparison of the goal task with the movement task. Indeed, relative to the goal task, the movement task showed a relative *de*-activation of the premotor/parietal movement identification regions, as well as the posterior temporal regions implicated in the visual analysis of motion (see Fig 2, panel C). Thus, while the goal task draws upon regions involved in object representations, the core nodes of the classical action observation/mirror network identified by the movement task are less in activated in the goal task.

### Whole brain parametric analysis of subjective action ratings

**Fig 3:**
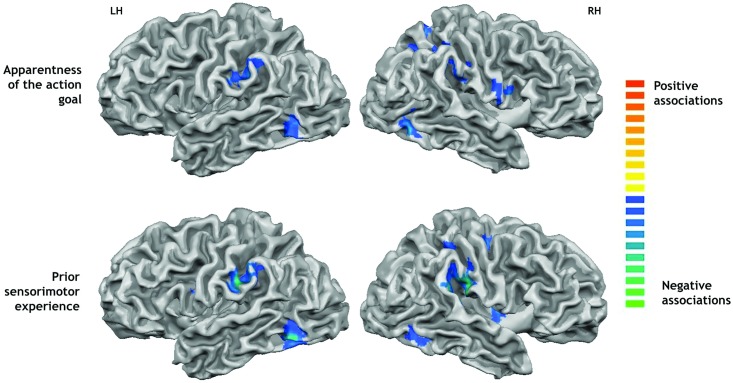
Parametric activations. Shows brain regions showing associations with the participants’ subjective action ratings. Top panel: negative associations with the apparentness of the action goals. Lower panel: negative associations with the amount of prior sensorimotor experience with the actions. All activations thresholded at *p* < 0.005 and whole-brain corrected to a familywise error *p* < 0.05.

The data from the post-scan questionnaires were used to correlate, on a single subject level, brain activity to the individual subjects’ ratings of the apparentness of the actions’ goals and the amount of sensorimotor experience they had with them (see Fig 3, Table 3). We first conducted a whole brain (corrected) analysis in which the results for goal apparentness and sensorimotor experience were collapsed across tasks (goal, object, movement, passive viewing) for maximum power. The analysis of goal apparentness revealed two regions in the right medial frontal gyrus, which were more activated the more apparent the goal of the presented actions was. Conversely, eight regions were activated more strongly for actions with non-apparent goals. They overlapped with regions identified by the movement task, such as the inferior parietal lobe/SMG bilaterally, as well as other motor and sensory areas such as the right precentral and postcentral gyrus, and visual areas such as the right occipitotemporal area, and the left fusiform gyrus and insula. Movement related regions therefore become activated specifically for actions in which the goals were less apparent.

**Table 3:** Whole brain activations varying parametrically with subjective action judgments.

Contrast	Region (BA)	R/L	x,y,z	t	mm^3^
***Apparentness of action goal***					
*Positive associations*	Medial Frontal Gyrus/Anterior Cingulate (32)	R	4,43,-4	6.34	1323
	Medial Frontal Gyrus (10)	R	8,59,15	5.12	567
*Negative associations*	Fusiform Gyrus (37)	L	-45,-63,-11	6.46	1782
	Inferior Parietal Lobe (40)	L	-51,-36,34	6.04	1431
	Superior Parietal Lobe (7)	R	12,-59,47	6.18	1809
	Occipitotemporal area (37)	R	51,-63,-7	5.87	1026
	Precentral Gyrus (6)	R	51,-1,31	5.31	864
	Postcentral Gyrus (2)	R	47,-27,33	4.63	837
	Insula (13)	L	-44,-7,7	5.07	648
	Inferior Parietal Lobe (40)	R	37,-43,47	4.43	567
***Sensorimotor experience***					
*Positive associations*	Fusiform Gyrus (37)	L	-27,-43,-10	4.57	1026
	Parahippocampal Gyrus (19)	L	-17,-54,-4	8.27	972
*Negative associations*	Inferior Parietal Lobe (40)	R	58,-27,23	7.72	3645
	Fusiform Gyrus (37)	L	-45,-61,-10	7.24	2565
	Inferior Parietal Lobe (40)	L	-57,-27,34	7.44	2511
	Fusiform Gyrus (37)	R	42,-52,-8	5.24	1431
	Insula (13)	R	34,-1,13	5.71	918
	Precentral Gyrus (6)	R	24,-16,55	4.98	810
	Medial Frontal Gyrus (6)	L	0,-19,48	5.35	675
	Inferior Frontal Gyrus (9)	L	-51,2,22	4.93	567

Correlations with ratings for sensorimotor experience revealed a similar pattern. They revealed positive activations in two regions in the left hemisphere, the fusiform gyrus and the parahippocampal gyrus, suggesting that these regions are activated in response to actions that have been performed often. Negative associations were again found in regions identified by the movement task, such as the bilateral inferior parietal lobe activation, as well as the precentral gyrus of the right hemisphere, the fusiform gyrus bilaterally, the right insula and the left inferior and medial frontal gyri.

### Region of interest analysis of subjective action ratings

The whole-brain analysis has suggested that regions involved in the motoric or visual analysis of action kinematics are recruited to a stronger extent when actions are unfamiliar or their goal is unclear. The same findings emerge when the parametric analyses are computed specifically for the regions of interest (ROIs, 15 mm cubes centred on the peak voxel) derived from the main contrast of movement vs. object task, which indexes the hypothesis-relevant regions involved in the motor/kinematic representation of action (see Supplementary Materials for results for regions derived from the other contrasts). Importantly, the increased power from the ROI analysis allows us to compute these comparisons for each of the four tasks separately (goal, movement, object, passive viewing). Because participants saw each action equally often in each task, the regressors for the subjective ratings used in this ROI analysis were statistically independent from the regressors used in identifying these regions, precluding artefactual inflation of statistical power due to non-independence (i.e. double-dipping, [48–49]).

This analysis revealed, averaged across all ROIs identified by the movement task, significant negative relationships between goal apparentness and sensorimotor experience specifically in the goal task (and the passive viewing task), but less so in the object and movement task (Table 4, bottom row). These negative relationships specifically for the goal task were also found when these relationships were tested for each region separately (Table 4). A 2 x 4 x 7 ANOVA with the factors rating, task and region revealed (greenhouse-Geisser corrected) main effects of task, *F*(3,39) = 3.25, *p* = .041, region, *F*(6,78) = 4.07, *p* = .009, as well as an interaction of region and rating, *F*(6,78) = 2.90, *p* = .036, showing that the different regions within the movement-related network responded differently to the two ratings, and that responses were most pronounced in the goal (and passive viewing) task compared to the object and movement tasks. These findings therefore show that regions involved in the kinematic/motoric analysis of actions are activated in the goal task, but particularly when goal identification is difficult, because observers have little experience with the actions that are unfamiliar or actions whose goals are unclear.

**Table 4:** Parametric regions from Movement task. Parametric trial-by-trial analyses of subjective action ratings (Exp., sensorimotor experience with the actions; App., apparentness of action goals), for each of the regions identified by the movement task (relative to the object task). The values show average across-participants beta values reflecting the relationship between individual participants’ ratings of the actions and brain activation while observing them in the four tasks.

Task	Movement		Object		Goal		Passive V.	
Region	App.	Exp.	App.	Exp.	App.	Exp.	App.	Exp.
Inferior Parietal Lobe R (40)	.05	.02	-.03	.01	-.04	-.05	-.06	-.01
Inferior Parietal Lobe L (40)	-.10	-.12	-.01	-.11	-.19	-.18**	-.19**	-.28***
Middle Temporal Gyrus R (37)	-.15**	-.15	-.13	-.19*	-.27**	-.32***	-.28***	-.31**
Middle Occipital Gyrus L (19)	-.08	-.05	.05	-.17	-.17***	-.28**	-.29***	-.36***
Cerebellum R	-.04	.07	.00	-.00	-.08	-.11	.05	.06
Superior Frontal Gyrus R (6)	-.00	.00	.07	-.00	-.19*	-.19**	-.11	-.09
Inferior Frontal Gyrus R (46)	.01	-.02	-.09	-.03	-.12	-.27***	-.07	-.05
**Overall**	**-.04**	**-.04**	**-.02**	**-.07**	-.15*	-.20***	-.14***	-.15**

* p < .10,

** p< .05,

*** p < .01.

## Discussion

Previous research has largely ignored the role of the objects and conceptualised action goal understanding primarily as a kinematic matching of observed actions to own motor representations. In contrast, our new data reveal that, at least for the everyday instrumental actions investigated here, action goal identification is more closely aligned—and shares large scale activation—with the processing of object semantics and function, instead of kinematic/motor processing. Indeed, our data suggest that the classical premotor-parietal “mirror” networks and temporal visual motion sensitive regions play a secondary role in action goal understanding and are recruited specifically when actions are unfamiliar or their goals are not clear.

Participants watched everyday instrumental actions and identified, in different tasks, either the movements performed (movement task), the objects used (object task), or the actions’ goals (goal task), while visual stimulation was kept identical. We tested, first, whether there are regions uniquely involved in action goal understanding, compared to both, motor/kinematic or object processing of the actions. Indeed, three left hemispheric regions—the inferior frontal gyrus, the middle temporal gyrus and the medial frontal gyrus—showed such a role, showing stronger activation in the goal task than in either the movement or objects tasks. Our data therefore confirms the proposed central role of these regions in action goal identification [12, 15, 50–51]. In addition, contra motoric theories of action understanding, they show for the first time that this role cannot be simply reduced to a motoric/kinematic (or object-based) encoding of the actions, instead revealing unique processes during action goal identification.

Second, and more importantly, our study revealed that, to derive these goals, the brain draws primarily on object-related information, rather than motor/kinematic information. Recall that the object task was designed to specifically recruit regions involved in object identification, not in the processing of body movements or the goals achieved with them. And indeed, relative to the movement task, it activated the expected network of left prefrontal and middle temporal regions previously implicated in encoding object semantics [31, 35, 52–53]. Importantly, the same regions were also activated when the *goal* task was compared to the movement task, demonstrating that goal understanding draws upon the same pre-frontal-temporal networks as object identification in the object task, in line with recent proposals that objects, because they are associated with specific functions, provide direct information about the goals that can be achieved with them [23, 25, 38]. In sharp contrast, testing for shared activations of goal and movement task revealed no such commonalities. Even though the movement task (relative to the object task) activated the classical set of “mirror” regions in premotor cortex and parietal lobe, as well as inferior temporal motion selective areas (for a review, see [54]), these regions were not found to be activated when the goal task was compared to the object task. In fact, direct comparisons revealed that these movement representation areas were *less* activated in the goal task than the movement task. Thus, while the goal task activates regions involved in object identification, it does not activate the motor/kinematic region networks engaged by the movement task.

These data are in line with prior reports noting an absence of classical mirror-like activations in higher-level goal understanding tasks (e.g., [14, 15]), and reveal for the first time that object identification—rather than motor or kinematic information—might provide the central input to goal inference processes. Note that the object task was explicitly designed to not require motor behaviour as a source of information, and that classic mirror or motion sensitive regions were relatively de-activated in the goal task. The contribution of object knowledge to goal identification is therefore unlikely to be motoric in nature. Instead, the overlap between the object and the goal task is consistent with the proposal that goal identification could specifically draw upon function knowledge about objects, such that knowledge about what an object is “for” can give the observed action its meaning [23, 25, 27, 38, 43, 26].

What, then, is the role of the classical ‘mirror’ regions? Our parametric analyses confirm that these regions do play a role in action goal understanding, but that they are recruited specifically when goal identification is difficult, when actions are unfamiliar and their goals are not clear. We correlated activity while viewing the different actions with participants’ ratings of how apparent their goal was and how much sensorimotor experience they had with them. Motoric matching theories predict that mirroring regions are specifically engaged whenever actions are familiar and can be matched to an action in one’s repertoire (e.g., [55]). However, in these studies, goal understanding was not required by the task and the actions did not involve objects, and therefore measured automatic responses of the mirror/motor system to the actions of others. Here, we found the reverse: when the goal of the action was clear and familiar, the anterior cingulate cortex (ACC) and parahippocampal gyrus, regions associated with mentalizing and memory became activated [56–57]. In contrast, premotor and parietal “mirroring” regions were specifically active when participants watched either unfamiliar actions or the action’s goals were ambiguous (for similar results, see [38, 44]). These results therefore confirm (1) that mirror and motion selective regions do play a specific role in action goal understanding, but (2) reveal that this contribution specifically concerns actions for which goals are not clear or unfamiliar. In addition, (3) focussed region of interest analysis tied these negative relationships directly to the goal task, not the motor task. The observed relationships are therefore unlikely to reflect automatic resonance of the motor system with familiar actions, but instead reflect a secondary recruitment of mirror and motion selective regions when action goals are task relevant but cannot be reliably inferred [38, 43]. Our findings are therefore in line with recent suggestions that these regions are not the primary driver of action goal attribution, but play a disambiguating or compensating role, for example, when action goals are unconstrained because object information is missing [24], when multiple actions (and goals) are possible in the given object context [25], or when visual input is uncertain, either due to ambiguous action input or disruption of visual processing itself [58, 59].

**Fig 4:**
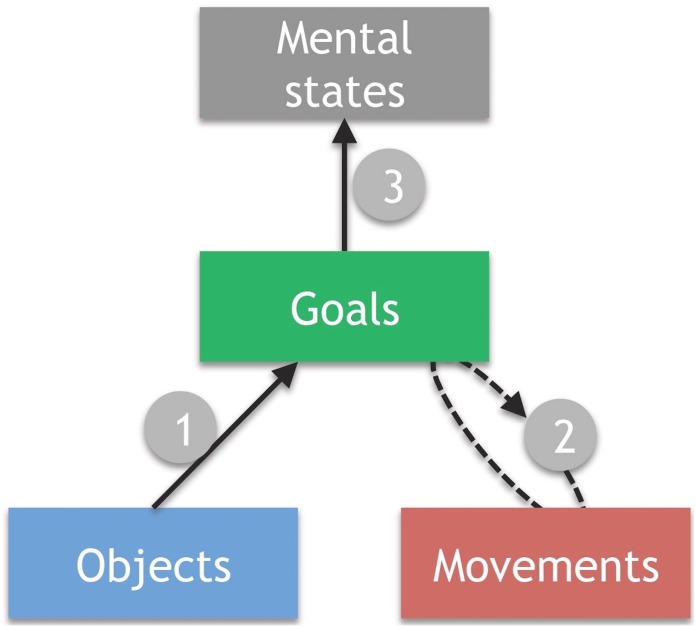
A simple model of everyday action understanding. Potential goals are initially identified on the basis of object information in inferior frontal and middle temporal areas (Step 1). Parietal-premotor motor-representation regions verify this initial interpretation or provide additional information (Step 2). If a goal is identified, associated mental states can in turn become activated—via medial prefrontal areas—and integrated with the situational context or prior knowledge about the person (Step 3).

Together, these data support previous arguments for a hierarchical model of action understanding [22, 23, 60], in which different action aspects—objects, movements, goals, and associated mental states—are represented separately (Fig 4). They specifically support models that argue for a major influence of object information in action goal understanding [23, 25, 27]. Such models suggest that for the type of instrumental action investigated here, potential action goals are initially derived from object function information, which, as found here, is localised to temporal and the inferior frontal regions (Path 1 in Fig 4). If a goal cannot be identified, because the actions are ambiguous or unfamiliar, premotor and parietal ‘mirror’ networks, as well as motion selective regions in the inferior temporal lobe, are recruited (Path 2). They provide a more detailed kinematic description of the movements, for example, to verify that the movements performed indeed conform to the assumed action goal, and that this goal will most likely be achieved [21, 25, 38, 43]. Finally (Path 3), the identification of a likely action goal allows for the action to be integrated with the situational context or with knowledge about the other person’s intentions, via medial prefrontal ‘mentalizing’ regions found in our study and others [14, 15].

While prior work has focussed on motor processes during action observation, more recent studies have started to delineate how objects constrain and guide action observation [22, 23], biasing perceptual processes [61], motor responses [1] and brain activations towards the expected action [25], as well as allowing different goals to be attributed to observed actions, even when the movements performed are identical [43, 45, 27]. Our new results are in line with these findings by showing direct overlaps between action goal understanding and the processing of object function and semantics. An open question is to what extend the current results generalise to other actions. Our set of actions was randomly selected from frequent actions of everyday life, with the only constraint that repetitions of objects, movements and goals across actions were controlled. It is therefore unlikely that the effects reflect specific processes associated with these particular actions. However, actions from other domains might differ in the extent to which they involve objects (jumping, scratching oneself, waving) and sometimes the relevant objects might not be visible (pantomimed actions, point light figures, objects hidden behind an occluder). These cases are likely to increase the requirement for accurate motor/kinematic action representations (for a review and theoretical view, see [23]), and further research needs to disentangle how the availability of object information constraints motor processes in a dynamic fashion [24, 25].

Similarly, the results will depend on the level of goals that is targeted. Action goals can be conceptualised in a hierarchical manner [18, 21, 60], where higher level task goals (e.g. turning on a light) depend on lower level motor goals (pressing down on a switch). The goals we investigated here were those higher-level task goals which describe “why” of an action [18]. Because such goals are often fully achieved only at the end of the action, or require further steps, they are typically more abstract and observers need to go beyond the information given by the stimulus to infer them [62]. Our data suggest that objects make a unique contribution here, probably because people learn from a very young achieve which function each object has and therefore which of these overarching goals it can help to achieve [23, 26]. The understanding of lower-level goals, in contrast, may depend to a larger extent on the specific motor and kinematic features of the movements on which they causally depend. Indeed, recent meta-analyses suggest that while over-arching task goals (the *why* of an action), like the ones studied here, activate similar inferior frontal and temporal regions as in our study, the tracking of more lower level motor goals relies more directly on motor-related regions in premotor and particularly parietal cortex [18, 51].

Another question is what types of processing the current activations reflect. On the highest level, the regions reflect the global requirements of the tasks to identify (and keep in working memory) the objects involved, coarse action kinematics and their goals. However, this might involve both regions for representing these action components, but also regions required to guide attention to these aspects, for example, tracking of motion in the movement task, and more static object-related processes in the object task. While our study was designed to test these global overlaps between tasks, such more specific distinctions between the roles of the different brain areas is subject to further research. Newer neuroimaging analysis methods such as repetition suppression and multivariate procedures [10, 12] will be able to provide direct information about these questions.

## Conclusions

We demonstrated a clear anatomical separation within the action observation network, between regions engaged in encoding the goal of an action on the one hand, and regions encoding the movement and objects on the other. We also demonstrate that, while goal processing is therefore strongly interwoven with object processing, movement-specific processes are only engaged in cases when the goal is unclear or participants have little experience with the observed actions. This does not rule out that actions are recognized if they can be matched to an action in one’s repertoire. However, it suggests that, for the perception of everyday object-directed actions, this matching process may occur primarily on the basis of the objects used rather than on the basis of kinematic information. These findings have direct implications for recent theories of action understanding and observation. Firstly, they highlight a left hemispheric frontotemporal (rather than frontoparietal) system in extracting action goals, which supports recent modelling work [22]. Secondly, they emphasise the role of visual cues, such as objects, rather than motor information, for the first step of action goal identification, as proposed by recent theories [23, 43]. Finally, it suggest kinematic action representations in parietal and premotor ‘mirror’ regions of the brain are not the main carrier of goal information, but that they are recruited by an alternative route if object information is diminished, perhaps to verify initial goal assumptions [21, 38, 43].

## Supporting Information

S1 Table Stimuli list. A list of all the video stimuli used in the experiment, along with the condition (and repetition) each clip was used for in relation to the participant’s task.
(DOCX)

S2 Table Parametric Region of Interest analysis. Combined Region of Interest analysis: Beta and p values for all main contrasts (across regions) correlated with ratings of the Apparentness of goal and Sensorimotor experience. Apparentness of action goal: Beta and p values for all Regions of Interest within each main contrast correlated with ratings of the apparentness of goal. Sensorimotor Experience: Beta and p values for all Regions of Interest within each main contrast correlated with ratings of sensorimotor experience.
(DOCX)
