# Supplementary material for: Understanding the Goals of Everyday Instrumental Actions Is Primarily Linked to Object, Not Motor-Kinematic, Information: Evidence from fMRI
Source: PLoS One. 2017 Jan 12;12(1):e0169700. doi: 10.1371/journal.pone.0169700 (PMC5231350; doi:10.1371/journal.pone.0169700)
Supplement: S1 Table — A list of all the video stimuli used in the experiment, along with the condition (and repetition) each clip was used for in relation to the participant’s task. (DOCX) [file pone.0169700.s001.docx]

**S1 Table. Stimuli list.** A list of all the video stimuli used in the experiment, along with the condition (and repetition) each clip was used for in relation to the participant’s task.

|  | **Title of Clip** | **Condition** | **Repetition** |
| --- | --- | --- | --- |
| 1 | Paying using card | Goal | Paying for something |
| 2 | Paying using cash | Goal | Paying for something |
| 3 | Drying hands using towel | Goal | Drying hands |
| 4 | Drying hands using hand dryer | Goal | Drying hands |
| 5 | Heating water on the hob | Goal | Heating water |
| 6 | Heating water with electric kettle | Goal | Heating water |
| 7 | Pulling drawstring | Goal | Making light |
| 8 | Switching light switch | Goal | Making light |
| 9 | Pressing play on ipod | Goal | Listening to music |
| 10 | Putting CD into stereo | Goal | Listening to music |
| 11 | Pouring water on instant coffee | Goal | Making coffee |
| 12 | Pushing plunger on cafferti | Goal | Making coffee |
| 13 | Knocking on door | Goal | Open door |
| 14 | Pressing doorbell | Goal | Open door |
| 15 | Putting clothes in washing machine | Goal | Wash clothes |
| 16 | Washing clothes in sink | Goal | Wash clothes |
| 17 | Turning microwave dial | Movement | Turning dial |
| 18 | Turning shower dial | Movement | Turning dial |
| 19 | Inserting baking tray in oven | Movement | Inserting |
| 20 | Inserting letter through letterbox | Movement | Inserting |
| 21 | Screwing lid on drink bottle | Movement | Screwing |
| 22 | Screwing light bulb in lamp | Movement | Screwing |
| 23 | Pressing pedestrian button | Movement | Pressing button |
| 24 | Pushing pushpin on notice board | Movement | Pressing button |
| 25 | Placing book in bag | Movement | 2 hand inserting |
| 26 | Placing computer in bag | Movement | 2 hand inserting |
| 27 | Rubbing sideboard with cloth | Movement | Rubbing |
| 28 | Rubbing wood with sandpaper | Movement | Rubbing |
| 29 | Pouring sugar in jar | Movement | Pouring |
| 30 | Pouring wine into glass | Movement | Pouring |
| 31 | Pulling blind | Movement | Pulling |
| 32 | Pulling drawstring toy | Movement | Pulling |
| 33 | Chopping carrot | Object | Knife |
| 34 | Cutting bread | Object | Knife |
| 35 | Screwing with screwdriver | Object | Screwdriver |
| 36 | Using screwdriver to remove staple | Object | Screwdriver |
| 37 | Stirring with spoon | Object | Spoon |
| 38 | Using spoon to put sugar on weetabix | Object | Spoon |
| 39 | Scraping egg from pan | Object | Spatula |
| 40 | Using spatula to stir | Object | Spatula |
| 41 | Using hammer to get nail out | Object | Hammer |
| 42 | Using hammer to hammer nail | Object | Hammer |
| 43 | Absorbing liquid with paper towel | Object | Paper towel |
| 44 | Cleaning glasses with paper towel | Object | Paper towel |
| 45 | Pricking potato with fork | Object | Fork |
| 46 | Whisking egg with fork | Object | Fork |
| 47 | Using pliers to cut wire | Object | Pliers |
| 48 | Using pliers to remove staple | Object | Pliers |
