# Supplementary material for: Understanding the Goals of Everyday Instrumental Actions Is Primarily Linked to Object, Not Motor-Kinematic, Information: Evidence from fMRI
Source: PLoS One. 2017 Jan 12;12(1):e0169700. doi: 10.1371/journal.pone.0169700 (PMC5231350; doi:10.1371/journal.pone.0169700)
Supplement: S2 Table — Combined Region of Interest analysis: Beta and p values for all main contrasts (across regions) correlated with ratings of the Apparentness of goal and Sensorimotor experience. Apparentness of action goal: Beta and p values for all Regions of Interest within each main contrast correlated with ratings of the apparentness of goal. Sensorimotor Experience: Beta and p values for all Regions of Interest within each main contrast correlated with ratings of sensorimotor experience. (DOCX) [file pone.0169700.s002.docx]

**S2 – Parametric Region of Interest analysis**

*Combined Region of Interest analysis - Beta and p values for all main contrasts (across regions) correlated with ratings of the Apparentness of goal and Sensorimotor experience*

| **Questionnaire** | **Contrast** | **Task** | | | | | | | |
| --- | --- | --- | --- | --- | --- | --- | --- | --- | --- |
|  |  | **Movement** | | **Object** | | **Goal** | | **Null** | |
|  |  | **Beta value** | **p** | **Beta value** | **p** | **Beta value** | **p** | **Beta value** | **p** |
| Apparentness | Object>Movement | 0.040 | 0.27 | 0.122 | 0.03** | -0.019 | 0.72 | -0.017 | 0.60 |
|  | Movement>Object | -0.044 | 0.29 | -0.020 | 0.59 | -0.15 | 0.03** | -0.136 | <0.01*** |
|  | Goal>Movement | 0.077 | <0.05** | 0.126 | 0.04** | 0.026 | 0.63 | 0.021 | 0.58 |
|  | Movement>Goal | -0.047 | 0.27 | -0.026 | 0.64 | -0.192 | 0.02** | -0.232 | <0.01*** |
|  | Goal>Object | 0.110 | 0.12 | 0.101 | 0.05** | -0.016 | 0.78 | -0.089 | 0.21 |
|  | Object>Goal | -0.013 | 0.75 | 0.015 | 0.86 | -0.151 | 0.03** | -0.156 | <0.01*** |
|  | Goal conjunction | 0.097 | 0.21 | 0.114 | 0.06* | -0.081 | 0.30 | -0.059 | 0.41 |
|  | Goal & Object Shared | 0.048 | 0.29 | 0.159 | 0.02** | 0.006 | 0.93 | 0.0002 | 1.00 |
| Experience | Object>Movement | 0.079 | 0.07* | 0.043 | 0.34 | -0.030 | 0.63 | -0.096 | 0.08* |
|  | Movement>Object | -0.036 | 0.45 | -0.070 | 0.14 | -0.198 | <0.01*** | -0.148 | 0.02** |
|  | Goal>Movement | 0.085 | 0.07* | 0.078 | 0.11 | -0.009 | 0.87 | -0.018 | 0.71 |
|  | Movement>Goal | -0.079 | 0.24 | -0.108 | 0.13 | -0.262 | <0.01*** | -0.258 | <0.01*** |
|  | Goal>Object | 0.062 | 0.29 | 0.044 | 0.33 | -0.107 | 0.12 | -0.115 | 0.06* |
|  | Object>Goal | -0.003 | 0.96 | -0.035 | 0.61 | -0.196 | 0.02** | -0.202 | 0.01** |
|  | Goal conjunction | 0.044 | 0.49 | 0.090 | 0.05** | -0.035 | 0.63 | -0.083 | 0.25 |
|  | Goal & Object Shared | 0.101 | 0.05** | 0.070 | 0.16 | -0.011 | 0.88 | -0.124 | 0.05** |

*Apparentness of action goal - Beta and p values for all Regions of Interest within each main contrast correlated with ratings of the apparentness of goal*

| **Contrast** | **Task** | | | | | | | | | | |
| --- | --- | --- | --- | --- | --- | --- | --- | --- | --- | --- | --- |
|  | **ROI** | **R/L** | **Peak**  **x,y,z** | **Movement** | | **Object** | | **Goal** | | **Null** | |
|  |  |  |  | **Beta** | **p** | **Beta** | **p** | **Beta** | **p** | **Beta** | **p** |
| Object>Movement | Cerebellum | R | 26,-80,-31 | -0.005 | 0.95 | 0.235 | 0.04** | 0.063 | 0.54 | 0.054 | 0.37 |
|  | Middle Temporal Gyrus (21) | L | -61,-38,-3 | 0.142 | 0.08* | 0.142 | 0.11 | -0.005 | 0.96 | 0.002 | 0.98 |
|  | Angular Gyrus (39) | L | -31,-65,33 | 0.047 | 0.40 | 0.185 | 0.03** | -0.092 | 0.38 | -0.022 | 0.62 |
|  | Fusiform Gyrus (37) | L | -28,-39,-18 | 0.139 | 0.03** | 0.114 | 0.10 | -0.031 | 0.73 | 0.028 | 0.71 |
|  | Inferior Frontal Gyrus (47) | L | -49,34,-6 | 0.058 | 0.44 | 0.174 | 0.01** | 0.094 | 0.39 | 0.062 | 0.33 |
|  | Parrahippocampal Gyrus (30) | L | -10,-50,6 | 0.268 | <0.01*** | 0.102 | 0.33 | 0.122 | 0.07* | 0.077 | 0.38 |
|  | Inferior Parietal Lobe (19) | R | 32,-68,42 | -0.047 | 0.45 | 0.087 | 0.39 | -0.141 | 0.07* | -0.029 | 0.58 |
|  | Inferior Frontal Gyrus (10) | L | -43,49,0 | -0.173 | 0.04** | 0.093 | 0.20 | -0.069 | 0.44 | -0.047 | 0.44 |
|  | Middle Frontal Gyrus (9) | L | -52,19,27 | -0.041 | 0.59 | 0.086 | 0.22 | -0.158 | 0.08* | -0.097 | 0.04** |
|  | Superior Frontal Gyrus (8) | L | -13,37,51 | -0.043 | 0.57 | -0.013 | 0.83 | -0.002 | 0.98 | -0.178 | 0.04** |
|  | Middle Occipital Gyrus (18) | L | -28,-86,2 | 0.093 | 0.26 | 0.133 | 0.24 | 0.006 | 0.94 | -0.032 | 0.70 |
| Movement>Object | Inferior Parietal Lobe (40) | R | -59,-44,24 | 0.053 | 0.43 | -0.034 | 0.70 | -0.039 | 0.64 | -0.064 | 0.21 |
|  | Inferior Parietal Lobe (40) | L | -59,-40,35 | -0.099 | 0.15 | -0.012 | 0.89 | -0.187 | <0.05** | -0.186 | 0.03** |
|  | Middle Temporal Gyrus (37) | R | 53,-56,-3 | -0.151 | 0.05** | -0.129 | 0.21 | -0.268 | <0.01*** | -0.283 | <0.01*** |
|  | Middle Occipital Gyrus (19) | L | -52,-68,-6 | -0.080 | 0.15 | 0.046 | 0.65 | -0.173 | 0.15 | -0.290 | <0.01*** |
|  | Cerebellum | R | 20,-44,-33 | -0.038 | 0.59 | 0.004 | 0.96 | -0.082 | 0.25 | 0.053 | 0.36 |
|  | Superior Frontal Gyrus (6) | R | 17,-8,-69 | -0.003 | 0.98 | 0.074 | 0.30 | -0.187 | 0.05* | -0.107 | 0.25 |
|  | Inferior Frontal Gyrus (46) | R | 50,31,11 | 0.013 | 0.90 | -0.090 | 0.15 | -0.116 | 0.34 | -0.073 | 0.30 |
| Goal>Movement | Middle Temporal Gyrus (21) | L | -61,-41,0 | 0.107 | 0.26 | 0.173 | 0.04** | -0.049 | 0.62 | 0.057 | 0.56 |
|  | Inferior Frontal Gyrus (47) | L | -49,31,-3 | 0.061 | 0.35 | 0.203 | <0.01*** | 0.063 | 0.58 | 0.042 | 0.51 |
|  | Superior/Medial Frontal Gyrus (9) | L | -14,49,29 | 0.046 | 0.48 | 0.170 | 0.04** | 0.020 | 0.80 | 0.038 | 0.63 |
|  | Angular Gyrus (39) | L | -34,-65,30 | 0.102 | 0.16 | 0.153 | 0.11 | 0.028 | 0.75 | 0.026 | 0.66 |
|  | Cerebellum | R | 17,-68,-30 | 0.033 | 0.64 | 0.147 | 0.16 | -0.032 | 0.74 | -0.063 | 0.42 |
|  | Posterior Cingulate (30) | L | -10,-56,11 | 0.229 | 0.01** | 0.135 | 0.17 | 0.165 | <0.05** | 0.060 | 0.48 |
|  | Posterior Cingulate (31) | L | -14,-50,27 | -0.004 | 0.96 | 0.073 | 0.37 | 0.131 | 0.21 | -0.079 | 0.45 |
|  | Fusiform Gyrus (37) | L | -28,-38,-18 | 0.154 | 0.05* | 0.129 | <0.10* | 0.020 | 0.81 | 0.012 | 0.88 |
|  | Medial Frontal Gyrus (10) | L | -4,49,-3 | 0.089 | 0.18 | 0.056 | 0.42 | 0.106 | 0.28 | 0.194 | 0.02** |
|  | Medial Frontal Gyrus (6) | L | -7,4,60 | 0.135 | 0.11 | 0.124 | 0.16 | -0.094 | 0.29 | -0.171 | <0.05** |
|  | Cerebellum | R | 35,-59,-31 | -0.042 | 0.62 | 0.081 | 0.56 | 0.036 | 0.67 | 0.081 | 0.37 |
|  | Superior Frontal Gyrus (10) | R | 32,49,11 | -0.054 | 0.55 | 0.063 | 0.39 | 0.045 | 0.52 | 0.058 | 0.45 |
|  | Fusiform Gyrus (37) | R | 17,-41,-9 | 0.151 | 0.03** | 0.130 | 0.25 | -0.099 | 0.27 | 0.022 | 0.83 |
| Movement>Goal | Inferior Temporal Gyrus (37) | R | 47,-68,0 | -0.096 | 0.10 | -0.022 | 0.85 | -0.183 | 0.08* | -0.188 | 0.01** |
|  | Inferior Temporal Gyrus (37) | L | -43,-68,0 | 0.001 | 0.98 | 0.006 | 0.96 | -164 | 0.10 | -0.315 | <0.01*** |
|  | Inferior Parietal Lobe (40) | L | -49,-38,33 | -0.032 | 0.63 | -0.145 | 0.03** | -0.191 | 0.02** | -0.196 | 0.02** |
|  | Superior Parietal Lobe (7) | R | 11,-68,45 | -0.061 | 0.24 | 0.012 | 0.84 | -0.217 | 0.04** | -0.238 | <0.01*** |
|  | Inferior Parietal Lobe/Supramarginal Gyrus (40) | R | 55,-35,36 | -0.024 | 0.77 | 0.024 | 0.69 | -0.223 | 0.05* | -0.251 | <0.01*** |
|  | Middle Frontal Gyrus (46) | R | 44,31,15 | -0.008 | 0.93 | -0.080 | 0.29 | -0.115 | 0.29 | -0.063 | 0.40 |
|  | Superior Parietal Lobe (7) | L | -19,-68,42 | -0.047 | 0.38 | 0.069 | 0.33 | -0.234 | 0.02** | -0.248 | <0.01*** |
|  | Inferior Parietal Lobe (40) | R | 35,-47,54 | -0.109 | 0.19 | -0.075 | 0.46 | -0.209 | <0.05** | -0.355 | <0.01*** |
| Goal>Object | Inferior Frontal Gyrus (45) | L | -52,22,6 | 0.084 | 0.34 | 0.138 | 0.06* | -0.054 | 0.59 | -0.072 | 0.43 |
|  | Middle Temporal Gyrus (39) | L | -49,-59,12 | 0.062 | 0.46 | 0.137 | 0.09* | 0.051 | 0.63 | -0.066 | 0.49 |
|  | Superior Frontal Gyrus (6) | L | -7,-5,62 | 0.148 | 0.04** | 0.073 | 0.37 | -0.059 | 0.47 | -0.171 | 0.11 |
|  | Anterior Cingulate Gyrus (32) | L | -1,25,24 | 0.147 | 0.13 | 0.057 | 0.47 | -0.002 | 0.98 | -0.045 | 0.64 |
| Object>Goal | Superior Parietal Lobe (7) | R | 29,-65,48 | -0.030 | 0.64 | 0.047 | 0.66 | -0.253 | <0.01*** | -0.184 | <0.01*** |
|  | Superior Parietal Lobe (7/19) | L | -25,-68,30 | 0.005 | 0.90 | 0.071 | 0.43 | -0.162 | 0.07* | -0.099 | 0.10 |
|  | Inferior Temporal Gyrus (37) | L | -49,-68,-3 | -0.035 | 0.63 | -0.031 | 0.78 | -0.226 | 0.04** | -0.291 | <0.01 |
|  | Lingual Gyrus (18) | L | -1,-86,2 | 0.009 | 0.89 | -0.026 | 0.83 | 0.037 | 0.66 | -0.050 | 0.50 |
| Goal conjunction | Inferior Frontal Gyrus (45) | L | -52,22,6 | 0.084 | 0.34 | 0.140 | 0.06* | -0.049 | 0.63 | -0.074 | 0.42 |
|  | Middle Temporal Gyrus (22) | L | -58,-41,3 | 0.061 | 0.58 | 0.124 | <0.05** | -0.081 | 0.43 | 0.062 | 0.52 |
|  | Medial Frontal Gyrus (6) | L | -5,1,60 | 0.146 | 0.07* | 0.08 | 0.36 | -0.112 | 0.21 | -0.165 | 0.06* |
| Goal & Object shared | Middle Temporal Gyrus (21) | L | -61,-38,-3 | 0.151 | 0.06* | 0.145 | 0.10 | -0.003 | 0.98 | 0.012 | 0.90 |
|  | Cerebellum | R | 22,-77,-27 | -0.026 | 0.74 | 0.197 | 0.08* | 0.02 | 0.84 | 0.039 | 0.49 |
|  | Inferior Frontal Gyrus (47) | L | -49,31,-3 | 0.075 | 0.28 | 0.199 | <0.01*** | 0.058 | 0.60 | 0.025 | 0.68 |
|  | Middle Frontal Gyrus (9) | L | -40,7,36 | -0.030 | 0.65 | 0.093 | 0.10 | -0.027 | 0.75 | -0.083 | 0.07* |
|  | Angular Gyrus (39) | L | -34,-65,33 | 0.070 | 0.28 | 0.163 | 0.08* | -0.019 | 0.85 | 0.008 | 0.90 |

*Sensorimotor Experience - Beta and p values for all Regions of Interest within each main contrast correlated with ratings of sensorimotor experience*

| **Contrast** | **Task** | | | | | | | | | | |
| --- | --- | --- | --- | --- | --- | --- | --- | --- | --- | --- | --- |
|  | **ROI** | **R/L** | **Peak**  **x,y,z** | **Movement** | | **Object** | | **Goal** | | **Null** | |
|  |  |  |  | **Beta** | **p** | **Beta** | **p** | **Beta** | **p** | **Beta** | **p** |
| Object>Movement | Cerebellum | R | 26,-80,-31 | 0.097 | 0.12 | 0.043 | 0.59 | -0.100 | 0.26 | -0.087 | 0.21 |
|  | Middle Temporal Gyrus (21) | L | -61,-38,-3 | 0.156 | 0.13 | 0.077 | 0.27 | 0.046 | 0.67 | -0.126 | 0.19 |
|  | Angular Gyrus (39) | L | -31,-65,33 | 0.102 | 0.16 | 0.032 | 0.69 | -0.017 | 0.85 | -0.132 | 0.12 |
|  | Fusiform Gyrus (37) | L | -28,-39,-18 | 0.097 | 0.23 | 0.174 | 0.03** | 0.043 | 0.66 | 0.035 | 0.6 |
|  | Inferior Frontal Gyrus (47) | L | -49,34,-6 | 0.057 | 0.45 | 0.091 | 0.18 | 0.070 | 0.47 | -0.174 | 0.06* |
|  | Parrahippocampal Gyrus (30) | L | -10,-50,6 | 0.267 | <0.01*** | 0.094 | 0.21 | 0.055 | 0.52 | 0.092 | 0.22 |
|  | Inferior Parietal Lobe (19) | R | 32,-68,42 | 0.085 | 0.23 | 0.044 | 0.66 | -0.072 | 0.32 | -0.058 | 0.53 |
|  | Inferior Frontal Gyrus (10) | L | -43,49,0 | -0.043 | 0.50 | 0.074 | 0.39 | -0.014 | 0.87 | -0.201 | <0.01*** |
|  | Middle Frontal Gyrus (9) | L | -52,19,27 | 0.004 | 0.94 | 0.088 | 0.26 | -0.169 | 0.05* | -0.169 | 0.04** |
|  | Superior Frontal Gyrus (8) | L | -13,37,51 | -0.051 | 0.66 | -0.189 | 0.04** | -0.053 | 0.51 | -0.048 | 0.57 |
|  | Middle Occipital Gyrus (18) | L | -28,-86,2 | 0.101 | 0.28 | -0.053 | 0.57 | -0.118 | 0.21 | -0.186 | 0.13 |
| Movement>Object | Inferior Parietal Lobe (40) | R | -59,-44,24 | 0.020 | 0.81 | 0.009 | 0.92 | -0.053 | 0.57 | -0.012 | 0.87 |
|  | Inferior Parietal Lobe (40) | L | -59,-40,35 | -0.118 | 0.16 | -0.111 | 0.24 | -0.176 | 0.04** | -0.277 | <0.01*** |
|  | Middle Temporal Gyrus (37) | R | 53,-56,-3 | -0.153 | 0.11 | -0.185 | 0.05* | -0.317 | <0.01*** | -0.311 | 0.02** |
|  | Middle Occipital Gyrus (19) | L | -52,-68,-6 | -0.052 | 0.54 | -0.173 | 0.11 | -0.277 | 0.03** | -0.361 | <0.01*** |
|  | Cerebellum | R | 20,-44,-33 | 0.072 | 0.45 | -0.001 | 0.99 | -0.108 | 0.24 | 0.062 | 0.31 |
|  | Superior Frontal Gyrus (6) | R | 17,-8,-69 | -0.001 | 0.99 | -0.002 | 0.97 | -0.190 | 0.04** | -0.091 | 0.21 |
|  | Inferior Frontal Gyrus (46) | R | 50,31,11 | -0.022 | 0.85 | -0.026 | 0.62 | -0.266 | <0.01*** | -0.045 | 0.56 |
| Goal>Movement | Middle Temporal Gyrus (21) | L | -61,-41,0 | 0.135 | 0.20 | 0.099 | 0.09* | 0.041 | 0.72 | -0.076 | 0.45 |
|  | Inferior Frontal Gyrus (47) | L | -49,31,-3 | 0.085 | 0.31 | 0.132 | <0.05** | 0.027 | 0.78 | -0.182 | 0.04** |
|  | Superior/Medial Frontal Gyrus (9) | L | -14,49,29 | -0.017 | 0.87 | -0.006 | 0.93 | -0.068 | 0.44 | 0.045 | 0.52 |
|  | Angular Gyrus (39) | L | -34,-65,30 | 0.186 | 0.03** | -0.024 | 0.79 | 0.017 | 0.85 | -0.098 | 0.30 |
|  | Cerebellum | R | 17,-68,-30 | 0.128 | <0.01*** | 0.082 | 0.29 | -0.128 | 0.06* | -0.102 | 0.23 |
|  | Posterior Cingulate (30) | L | -10,-56,11 | 0.184 | 0.04** | 0.129 | 0.09* | 0.060 | 0.30 | 0.022 | 0.77 |
|  | Posterior Cingulate (31) | L | -14,-50,27 | -0.064 | 0.54 | -0.007 | 0.91 | -0.003 | 0.96 | 0.006 | 0.94 |
|  | Fusiform Gyrus (37) | L | -28,-38,-18 | 0.153 | 0.08* | 0.216 | 0.01** | 0.108 | 0.29 | 0.066 | 0.30 |
|  | Medial Frontal Gyrus (10) | L | -4,49,-3 | 0.080 | 0.40 | 0.055 | 0.29 | 0.079 | 0.34 | 0.051 | 0.59 |
|  | Medial Frontal Gyrus (6) | L | -7,4,60 | -0.055 | 0.50 | 0.093 | 0.29 | -0.089 | 0.23 | -0.073 | 0.22 |
|  | Cerebellum | R | 35,-59,-31 | 0.126 | 0.14 | 0.044 | 0.75 | -0.083 | 0.31 | -0.014 | 0.87 |
|  | Superior Frontal Gyrus (10) | R | 32,49,11 | 0.072 | 0.46 | 0.022 | 0.73 | -0.146 | 0.07* | -0.025 | 0.77 |
|  | Fusiform Gyrus (37) | R | 17,-41,-9 | 0.091 | 0.28 | 0.175 | <0.10* | 0.066 | 0.45 | 0.152 | 0.12 |
| Movement>Goal | Inferior Temporal Gyrus (37) | R | 47,-68,0 | -0.060 | 0.53 | -0.150 | 0.14 | -0.221 | 0.04** | -0.300 | 0.01** |
|  | Inferior Temporal Gyrus (37) | L | -43,-68,0 | -0.039 | 0.65 | -0.255 | 0.02** | -0.336 | <0.01*** | -0.472 | <0.01*** |
|  | Inferior Parietal Lobe (40) | L | -49,-38,33 | -0.066 | 0.43 | -0.134 | 0.12 | -0.197 | 0.02** | -0.357 | <0.01*** |
|  | Superior Parietal Lobe (7) | R | 11,-68,45 | -0.075 | 0.32 | -0.046 | 0.63 | -0.148 | 0.18 | -0.187 | 0.01** |
|  | Inferior Parietal Lobe/Supramarginal Gyrus (40) | R | 55,-35,36 | -0.175 | 0.12 | -0.077 | 0.40 | -0.317 | <0.01*** | -0.133 | 0.03** |
|  | Middle Frontal Gyrus (46) | R | 44,31,15 | -0.082 | 0.47 | -0.036 | 0.57 | -0.314 | <0.01*** | -0.057 | 0.48 |
|  | Superior Parietal Lobe (7) | L | -19,-68,42 | -0.018 | 0.83 | 0.013 | 0.88 | -0.182 | 0.09* | -0.252 | <0.01*** |
|  | Inferior Parietal Lobe (40) | R | 35,-47,54 | -0.113 | 0.22 | -0.179 | 0.16 | -0.383 | <0.01*** | -0.309 | <0.01*** |
| Goal>Object | Inferior Frontal Gyrus (45) | L | -52,22,6 | 0.100 | 0.27 | 0.086 | 0.18 | -0.074 | 0.38 | -0.166 | 0.02** |
|  | Middle Temporal Gyrus (39) | L | -49,-59,12 | 0.090 | 0.26 | 0.038 | 0.63 | -0.106 | 0.28 | -0.102 | 0.30 |
|  | Superior Frontal Gyrus (6) | L | -7,-5,62 | -0.011 | 0.89 | 0.026 | 0.76 | -0.120 | 0.13 | -0.187 | 0.03** |
|  | Anterior Cingulate Gyrus (32) | L | -1,25,24 | 0.068 | 0.52 | 0.026 | 0.67 | -0.127 | 0.17 | -0.006 | 0.91 |
| Object>Goal | Superior Parietal Lobe (7) | R | 29,-65,48 | 0.035 | 0.61 | 0.028 | 0.79 | -0.186 | 0.04** | -0.225 | 0.03** |
|  | Superior Parietal Lobe (7/19) | L | -25,-68,30 | 0.056 | 0.29 | 0.120 | 0.16 | -0.093 | 0.22 | -0.124 | 0.14 |
|  | Inferior Temporal Gyrus (37) | L | -49,-68,-3 | -0.103 | 0.21 | -0.292 | <0.01*** | -0.364 | <0.01*** | -0.474 | <0.01*** |
|  | Lingual Gyrus (18) | L | -1,-86,2 | 0.002 | 0.99 | 0.003 | 0.97 | -0.140 | 0.11 | 0.015 | 0.82 |
| Goal conjunction | Inferior Frontal Gyrus (45) | L | -52,22,6 | 0.099 | 0.28 | 0.088 | 0.17 | -0.057 | 0.51 | -0.163 | 0.02** |
|  | Middle Temporal Gyrus (22) | L | -58,-41,3 | 0.087 | 0.39 | 0.116 | 0.02** | 0.032 | 0.76 | -0.02 | 0.85 |
|  | Medial Frontal Gyrus (6) | L | -5,1,60 | -0.053 | 0.52 | 0.066 | 0.47 | -0.079 | 0.26 | -0.065 | 0.32 |
| Goal & Object shared | Middle Temporal Gyrus (21) | L | -61,-38,-3 | 0.155 | 0.14 | 0.077 | 0.27 | 0.047 | 0.67 | -0.126 | 0.19 |
|  | Cerebellum | R | 22,-77,-27 | 0.075 | 0.20 | 0.059 | 0.47 | -0.074 | 0.34 | -0.098 | 0.21 |
|  | Inferior Frontal Gyrus (47) | L | -49,31,-3 | 0.083 | 0.33 | 0.120 | 0.07* | 0.031 | 0.75 | -0.185 | 0.04** |
|  | Middle Frontal Gyrus (9) | L | -40,7,36 | 0.071 | 0.27 | 0.114 | 0.09* | -0.083 | 0.40 | -0.083 | 0.26 |
|  | Angular Gyrus (39) | L | -34,-65,33 | 0.120 | 0.15 | -0.020 | 0.79 | 0.025 | 0.78 | -0.127 | 0.13 |
